# Standard versus Abdominal Lifting and Compression CPR

**DOI:** 10.1155/2016/9416908

**Published:** 2016-11-01

**Authors:** Sisen Zhang, Qing Liu, Shupeng Han, Ziran Zhang, Yan Zhang, Yahua Liu, Jing Li, Lixiang Wang

**Affiliations:** ^1^Department of Emergency, Southern Medical University Affiliated Zhengzhou People's Hospital, Zhengzhou, Henan 450003, China; ^2^Emergency Medical Center, General Hospital of Chinese Armed Police Forces, Beijing 100039, China; ^3^Beijing Germari Medical Equipment Co., Ltd, Beijing, China

## Abstract

*Background*. This study compared outcomes of abdominal lifting and compression cardiopulmonary resuscitation (ALP-CPR) with standard CPR (STD-CPR).* Materials and Methods*. Patients with cardiac arrest seen from April to December 2014 were randomized to receive standard CPR or ALP-CPR performed with a novel abdominal lifting/compression device. The primary outcome was return of spontaneous circulation (ROSC).* Results*. Patients were randomized to receive ALP-CPR (*n* = 40) and STD-CPR (*n* = 43), and the groups had similar baseline characteristics. After CPR, 9 (22.5%) and 7 (16.3%) patients in the ALP-CPR and STD-CPR groups, respectively, obtained ROSC. At 60 minutes after ROSC, 7 (77.8%) and 2 (28.6%) patients, respectively, in the ALP-CPR and STD-CPR groups survived (*P* = 0.049). Patients in the ALP-CPR group had a significantly higher heart rate and lower mean arterial pressure (MAP) than those in the STD-CPR group (heart rate: 106.8 versus 79.0, *P* < 0.001; MAP: 60.0 versus 67.3 mm Hg, *P* = 0.003). The posttreatment PCO_2_ was significantly lower in ALP-CPR group than in STD-CPR group (52.33 versus 58.81, *P* = 0.009). PO_2_ was significantly increased after ALP-CPR (45.15 to 60.68, *P* < 0.001), but it was not changed after STD-CPR. PO_2_ after CPR was significantly higher in the ALP-CPR group (60.68 versus 44.47, *P* < 0.001). There were no differences between genders and for patients who are > 65 or ≤ 65 years of age.* Conclusions*. The abdominal lifting and compression cardiopulmonary resuscitation device used in this study is associated with a higher survival rate after ROSC than standard CPR.

## 1. Introduction

Even when immediate cardiopulmonary resuscitation (CPR) is administered after cardiac arrest, the restoration of spontaneous circulation (ROSC) success rate has remained relatively low [1, 2]. As a result, research has been devoted to developing alternatives to conventional CPR to improve the resuscitation success rate [3]. Tang et al. [4] reported that phased chest and abdominal compression-decompression substantially increased hemodynamic efficacy of CPR and outcome in terms of successful resuscitation, 48-hour survival, and cerebral recovery. Aliverti et al. [5] suggested on the basis of their research that the abdomen functions as the body's “second heart” during cardiac arrest. And Sack et al. studied that, in 135 resuscitation attempts in 103 patients experiencing in-hospital cardiac arrest during a 6-month period, the results provided clear mechanism for the abdominal resuscitation [6]. Nevertheless, the foregoing studies all focused on the abdominal compression process and ignored the effect of abdominal lifting.

Abdominal lifting and compression cardiac resuscitation devices can perform active compression and lifting based on the “thoracic pump,” [7] “abdominal pump,” [8] and “heart pump” [9] mechanisms. The idea of three pumps and the thoracic, abdominal, and cardiac pump mechanisms is classically known in the literature from the decade of the 1980s [7]. The devices employ abdominal lifting and compression to induce pressure changes in the abdominal cavity, which activates the “abdominal pump.” The piston effect of the diaphragm in the thoracic and abdominal cavities then transmits pressure changes in the abdominal cavity to the thoracic cavity, inducing thoracic pressure changes which indirectly activate the “thoracic pump.” The anatomical relationship of the heart and diaphragm then activates the “heart pump,” which results in blood flow. And an advantage of abdominal pumping on the chest is that it would promote some ventilation [8]. Animal experiments using this method have demonstrated significant effectiveness [10, 11].

The purpose of this study was to compare the outcomes of an abdominal lifting and compression device with those of conventional CPR in patients with cardiopulmonary arrest.

## 2. Materials and Methods

### 2.1. Ethical Approval

The effectiveness, safety, and stability of the abdominal lifting and compression device used in this study have been verified in animal and human experiments [12]. This study was approved by the Ethical Review Committee of Zhengzhou People's Hospital. All patient relatives and legal guardians received a detailed explanation of the study's possible risks and benefits and were permitted to request discontinuation of the study at any time. The requirements of the Declaration of Helsinki were strictly upheld throughout the research process.

### 2.2. Patients

This was a prospective study conducted at Zhengzhou People's Hospital from April to December 2014. Adults of both genders with a body weight of 40–150 kg meeting American Heart Association (AHA) guidelines for cardiopulmonary arrest seen in the emergency department were eligible for inclusion [8]. The criteria include (a) loss of consciousness, (b) loss of heart sound and pulse in the carotid and femoral artery, (c) sighing respiration, and (d) pupil dilation and weakening, or disappearance of response to light. In addition, it was required that a close relative or legal guardian of the patient provide written informed consent to participate in the study. Exclusion criteria were (1) no indication for resuscitation or a do not resuscitate order; (2) contraindication to the use of abdominal lifting and compression (contraindications include external injury to the abdomen, rupture of the diaphragm, bleeding in the abdominal cavity or internal organs, abdominal aortic aneurysm, and large tumor in the abdominal cavity) or injury to the abdominal cavity or internal organs during abdominal compression; (3) disease that might significantly affect assessment of effectiveness (e.g., chronic wasting diseases such as malignancy or severe tuberculosis); and (4) informed consent not obtained.

### 2.3. Interventions

Abdominal lifting and compression CPR (ALP-CPR) and standard CPR (STD-CPR) were used to treat patients in accordance with a random number table generated using SPSS 20.0 software. Numbers from the random number table were assigned on a unified basis by the hospital's emergency center dispatching department. All patients received orotracheal intubation, respiration with the aid of a rebreathing bag, and electrocardiograph monitoring. Two intravenous lines were established and rapid infusion of 250 mL × 2 of 0.9% sodium chloride solution was given. Defibrillation was administered as needed. All personnel providing care were trained in advanced CPR techniques and the use of the abdominal lifting/compression device.

**Figure 1:**
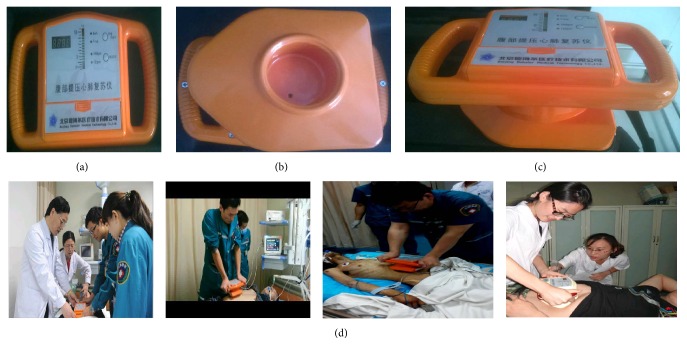
(a–c) CPR-LW1000 abdominal lifting and compression device. (d) Device in use.

A model CPR-LW1000 abdominal lifting/compression device invented by Professor Wang Lixiang of the General Hospital of Armed Police Forces' Emergency Medical Center and produced by the Beijing Germari Medical Equipment Co., Ltd., was used to perform ALP-CPR [11]. The device is composed of three components: a display panel, pressure application handles, and a negative pressure device. The instrument is operated holding the pressure application handles and placing the compression plate on the patient's abdomen. After turning on the device, negative pressure is generated which causes a tight bond between these pressure plates and the patient's abdomen. The operator then presses an indicator light prompted by an audio signal with a frequency of 100 times/minute, and the instrument performs alternate vertical downward compressions and upward lifting actions. The duration of compression and lifting was performed in a 1 : 1 ratio, the pressure was approximately 186 mmHg when the indicator light was on, and lifting force was approximately 112 mmHg. Images of the device are shown in Figure 1.

### 2.4. Termination of Lifesaving Treatment

In compliance with AHA guidelines, lifesaving treatment was considered successful and terminated with the appearance of an autonomous aortic pulse, moist facial complexion, the appearance of autonomous respiration, and shrinking pupils and reappearance of a light reflex, or the appearance of eyeball motion and limb spasms [13]. If after continued routine lifesaving efforts for at least 30 minutes no pulse or autonomous breathing was noted, lifesaving treatment was terminated after obtaining informed consent from family members.

### 2.5. Outcome Measures

The primary outcome measure was ROSC rate (restoration of sinus or supraventricular rhythm, mean arterial pressure (MAP) ≥ 50–60 mm Hg, maintained for ≥20 minutes). Secondary outcome measures were blood pressure, heart rate, blood gas parameters, and MAP before, during, and after patient resuscitation. Viability at 30 and 60 minutes after ROSC was also recorded.

### 2.6. Statistical Analysis

The primary endpoint, ROSC, was presented by number and percentage, and the difference of ROSC rate between the two CPR groups was tested with the two-proportion *Z*-test. Continuous variables were presented by mean and standard deviation, and differences between the two groups were tested with the independent two-sample *t*-test, and changes from baseline to after CPR within groups were tested with the paired *t*-test. Sex of the two groups was presented by number and percentage, and differences were tested with Fisher's exact test. Values of *P* < 0.05 were considered to indicate statistical significance. All analyses were performed using SPSS 22 statistical software (IBM Corp., Armonk, NY, USA).

### 2.7. Sample Size

According to the equation below, at least 45 subjects were required in each group to detect a difference of ROSC rate between the ALP-CPR and STD-CPR groups with the power of 0.8  (1 − *β*) and a significance level of 0.05 (*α*).

Equation:

(1) $$\begin{array}{c} n_{1}=n_{2}=\frac{{\left(z_{\alpha /2}+z_{\beta }\right)}^{2}\left[p_{1}\left(1-p_{1}\right)+p_{2}\left(1-p_{2}\right)\right]}{{\left(p_{1}-p_{2}\right)}^{2}}, \end{array}$$

where *p*_1_ and *p*_2_ were set at 0.21 [14] and 0.48 [15].

## 3. Results

### 3.1. Patients

**Figure 2:**
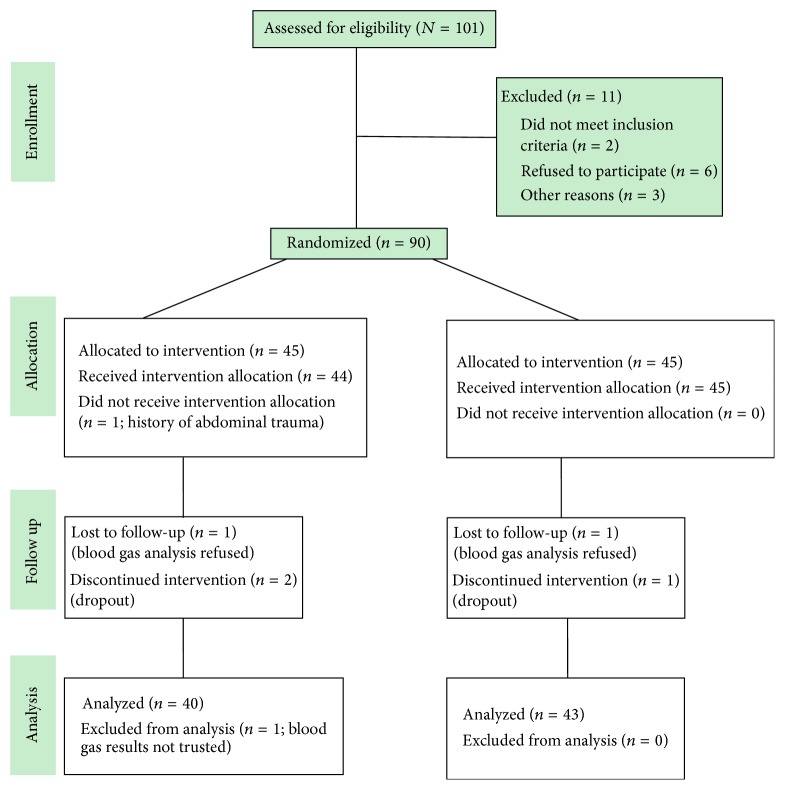
Patient enrollment and disposition.

A flow diagram of patient selection and disposition is shown in Figure 2. Of 101 patients initially screened, 90 were randomized to the two groups and ultimately data of 40 and 43 patients in the ALP-CPR and STD-CPR groups, respectively, were available for analysis.

Patients in the two groups had comparable baseline characteristics (Tables 1 and 2).

### 3.2. ROSC

After CPR, 9 (22.5%) and 7 (16.3%) patients in the ALP-CPR and STD-CPR groups, respectively, obtained ROSC. At 30 minutes after ROSC, 7 (77.8%) and 4 (57.1%) patients, respectively, in ALP-CPR and STD-CPR groups survived and the difference did not reach statistical significance. At 60 minutes after ROSC, 7 (77.8%) and 2 (28.6%) patients, respectively, in the ALP-CPR and STD-CPR groups survived, and the survival rate was significantly higher in the ALP-CPR group (*P* = 0.049) (Table 3).

### 3.3. Vital Signs

After CPR, nearly all patients obtained heart rate and MAP recovery, but only 13 in the ALP-CPR and 12 in the STD-CPR obtained recovery of respiration. Patients in the ALP-CPR group had a significantly higher heart rate and lower MAP than those in the STD-CPR group (heart rate: 106.8 versus 79.0, *P* < 0.001; MAP: 60.0 versus 67.3 mmHg, *P* = 0.003). The respiration rate of the two groups after CPR was not significantly different. At 30 minutes after ROSC, the 7 patients in ALP-CPR group had a significantly higher heart rate than the 4 patients in STD-CPR group (Table 3).

### 3.4. Change of Blood Gas Measurements

The blood pH levels of the two groups were comparable at baseline and then decreased after CPR (all, *P* < 0.001), and the pH in the ALP-CPR group was reduced to a greater degree than in the STD-CPR group (−0.16 versus −0.09, *P* = 0.037) (Table 2). The pH after CPR of the ALP-CPR group was significantly lower than that of the STD-CPR group (7.06 versus 7.17, *P* = 0.005).

SPO_2_ showed no significant change after ALP-CPR, but it was significantly increased after STD-CPR (39.65 to 54.21, *P* = 0.010). PCO_2_ was significantly decreased after ALP-CPR (57.20 to 52.33, *P* = 0.012), but it was not changed after STD-CPR. The posttreatment PCO_2_ was significantly lower in the ALP-CPR than in STD-CPR group (52.33 versus 58.81, *P* = 0.009). PO_2_ was significantly increased after ALP-CPR (45.15 to 60.68, *P* < 0.001) but was not changed after STD-CPR. PO_2_ after CPR was significantly higher in the ALP-CPR group than in the STD-CPR group (60.68 versus 44.47, *P* < 0.001).

The ALP-CPR group had a higher K^+^ and lower Ca^2+^ level compared to the STD-CPR group at baseline, but compared to baseline levels no significant change of K^+^ and Ca^2+^ was observed after CPR. The LAC levels of the two groups were comparable at baseline, and then both decreased after CPR (both, *P* < 0.001), but the reduction was less in the ALP-CPR than in the STD-CPR group (−0.43 versus −1.32, *P* < 0.001) (Table 2).

### 3.5. Associations of Sex and Age with Vital Signs and Changes of Blood Gas Measurements

There was no significant difference between males and females with respect to vital signs and changes of blood gas measurements after ALP-CPR. For STD-CPR, there was also no significant difference between males and females, except for respiration rate: males had a significantly lower respiration rate than females after STD-CPR (15.33 versus 25.33, *P* < 0.001) (Table 4).

For patients who received ALP-CPR, there were no significant differences of vital signs and changes of blood gas measurements between those who are >65 years and ≤65 years of age, except for K^+^ (change from baseline: −0.52 versus 0.29, *P* = 0.031). For the STD-CPR patients, there were no significant differences of vital signs and changes of blood gas measurements between those who are >65 years and ≤65 years of age, except for Ca^2+^ (change from baseline: −012 versus 0.06, *P* = 0.049) (Table 5).

## 4. Discussion

The results of this study showed that while ROSC was comparable between the two groups, survival after ROSC was significantly better in the ALP-CPR than the STD-CPR group (77.8% versus 28.6%). Significant changes in blood gas measurements were observed, and outcomes were not affected by sex or age.

After more than 50 years of investigation and practice, although the ROSC rate has increased, the resuscitation success rate of CPR remains inadequate [1, 2]. Chest compressions are contraindicated in some situations, rib fractures may occur in 1/3 of cases, and increasing compression depth increases the complication rates [13, 16].

Abdominal lifting and compression CPR is a new technology that generates artificial circulation and ventilation via the thoracic, abdominal, and heart pump mechanisms [10]. The instrument used in this study has an abdominal contact area of approximately 200 cm^2^. After negative pressure results in abdominal suction, the user operates the instrument in accordance with the display screen and the audio signals. The compression force is 40–50 kg, which is equivalent to pressure of 1.96–2.45 kgf/cm^2^ on the abdominal wall (1.90–2.37 atmospheres) [5]. Each instance of compression causes approximately 300 mL of blood to enter the effective circulation. During abdominal lifting, pressure within the abdominal cavity decreases causing the femoral vein to open allowing venous blood from the legs to enter the internal organs [5]. At the same time, decreased pressure within the abdominal cavity causes the diaphragm to fall back, the volume of the thoracic cavity increases and the pressure drops, and the heart enters a diastolic state with subsequent blood flow into the heart which prepares the heart for the next compression [17]. In addition, adequate coronary perfusion pressure (CPP) is important for successful CPR, and abdominal compression can significantly increase CPP [14].

In most cases of primary cardiopulmonary arrest, blood still contains some oxygen during the early period. As a result, the reduction in oxygen to the myocardium and brain is chiefly due to reduced circulation and not to reduced ventilation or a drop in blood oxygen, which is why the restoration of circulation is emphasized during the early stage of resuscitation [18]. However, the prognosis after CPR is still not ideal [15]. In cases of cardiac arrest and subsequent lifesaving treatment at general hospitals in the United States, 61.5% of patients die before hospital discharge and, of these, 46.0% die of injuries to the nervous system and over 20% of surviving patients suffer permanent functional impairment of the nervous system [19]. This suggests that conventional chest compression is not able to provide optimal brain perfusion. Subdiaphragmatic cardiac compression will cause blood in the abdominal aorta to flow in reverse, which increases perfusion pressure in the heart, brain, and other important organs [11]. Furthermore, when pressure is directly applied to the abdominal aorta, the pressure difference between the central arteries and veins reaches a maximum which can significantly increase perfusion of the heart and brain.

Large-scale study has reported that compression-only CPR or STD-CPR are equivalent with respect to prognosis and ROSC rate [20]. However, other research has indicated that the vast majority of cardiac arrest cases result from choking [21], and improvement of ventilation is necessary for ROSC. While the 2010 AHA guidelines stress the importance of chest compression, the emphasis on the continuity of circulation does not imply that we should ignore the need of early ventilation support and compression alone cannot achieve adequate ventilation. However, abdominal lifting and compression results in upward and downward motion of the diaphragm and pressure changes in the thorax. The downward motion of the diaphragm increases the negative pressure in the thorax at which time air enters the lungs, and the downward motion of the diaphragm facilitates discharge of air from the lungs. Pargett et al. [22] showed that rhythmic abdominal compression CPR ventilates without supplemental breaths and provides effective blood circulation.

While there were some statistically significant differences in baseline K^+^ and Ca^2+^ levels between the groups, this was not of clinical significance to this study; as for both groups, the K^+^ and Ca^2+^ levels were not excessive to have impacted heart resuscitation.

There are limitations to this study that should be considered. The study was performed at a single center, and the number of patients was limited. Due to the limited sample and the fact that most cases of cardiac arrest occur among older patients, age groups younger than 65 years were not examined. Autopsies were not performed in nonsurvivors and thus we were not able to determine if abdominal lifting and compression resulted in abdominal injuries.

## 5. Conclusions

The abdominal lifting and compression cardiopulmonary resuscitation device used in this study is associated with a higher survival rate after ROSC than standard CPR. The device is recommended for use in the lifesaving treatment of cardiac arrest patients who have contraindications against standard chest compression. However, we also have some limitations that no autopsy data on abdominal damage are available. There might has been damage with peak pressure of over 760 mmHg.

## Figures and Tables

**Table 1:** Patient baseline characteristics.

	ALP-CPR (*n* = 40)	STD-CPR (*n* = 43)	*P* value
Sex			
Female	23 (57.5%)	22 (51.2%)	0.148
Male	17 (42.5%)	21 (48.8%)	
Age (y)	64.9 (14.9)	62.5 (13.7)	0.450
Cardiac arrest time (min)	8.0 (3.1)	8.8 (8.0)	0.574
Weight (kg)	65.1 (9.9)	64.1 (10.2)	0.634
Height (cm)	163.3 (10.4)	161.0 (9.3)	0.293
BMI (kg/m^2^)	24.5 (3.2)	24.8 (4.0)	0.700

Data are presented as mean (standard deviation) or number and percentage.

ALP-CPR, abdominal lifting and compression cardiopulmonary resuscitation; BMI, body mass index; STD-CPR, standard CPR.

**Table 2:** Blood gas and electrolyte measurements before and after CPR.

		ALP-CPR (*n* = 40)	STD-CPR (*n* = 43)	*P* value
pH	Baseline	7.22 (0.19)	7.25 (0.16)	0.397
	After CPR	7.06 (0.18)^†^	7.17 (0.17)^†^	0.005^*∗*^
	Change from baseline	−0.16 (0.18)	−0.09 (0.15)	0.037^*∗*^

SPO_2_	Baseline	33.25 (28.03)	39.65 (22.02)	0.249
	After CPR	44.00 (34.15)	54.21 (34.76)^†^	0.181
	Change from baseline	10.75 (48.58)	14.56 (35.55)	0.687

PCO_2_	Baseline	57.20 (7.42)	57.63 (11.26)	0.838
	After CPR	52.33 (9.07)^†^	58.81 (12.57)	0.009^*∗*^
	Change from baseline	−4.88 (11.74)	1.19 (17.83)	0.070

PO_2_	Baseline	45.15 (7.76)	45.33 (18.36)	0.954
	After CPR	60.68 (12.96)^†^	44.47 (23.94)	<0.001^*∗*^
	Change from baseline	15.53 (15.10)	−0.86 (29.76)	0.002^*∗*^

K^+^	Baseline	5.02 (0.91)	3.99 (0.77)	<0.001^*∗*^
	After CPR	4.90 (1.04)	3.98 (0.81)	<0.001^*∗*^
	Change from baseline	−0.11 (1.20)	−0.02 (1.00)	0.694

Ca^2+^	Baseline	1.32 (0.44)	2.01 (0.22)	<0.001^*∗*^
	After CPR	1.65 (1.13)	1.99 (0.29)	0.080
	Change from baseline	0.34 (1.06)	−0.03 (0.31)	0.044^*∗*^

LAC	Baseline	5.91 (1.63)	5.79 (1.47)	0.711
	After CPR	5.49 (1.40)^†^	4.47 (0.98)^†^	<0.001^*∗*^
	Change from baseline	−0.43 (0.54)	−1.32 (1.11)	<0.001^*∗*^

Data are presented as mean (standard deviation).

ALP-CPR, abdominal lifting and compression cardiopulmonary resuscitation; STD-CPR, standard CPR; LAC, lactate.

__^*∗*^ *P* < 0.05 indicates a significant difference between ALP-CPR and STD-CPR groups.

__^†^ *P* < 0.05 indicates a significant change from baseline within group.

**Table 3:** ROSC rate and vital signs.

		ALP-CPR (*n* = 40)	STD-CPR (*n* = 43)	*P* value
ROSC	After CPR	9/40 (22.5%)	7/43 (16.3%)	0.473
	Survived 30 minutes after ROSC	7/9 (77.8%)	4/7 (57.1%)	0.377
	Survived 60 minutes after ROSC	7/9 (77.8%)	2/7 (28.6%)	0.049^*∗*^

Heart rate (beats/min)	After CPR	106.8 (9.3), *n* = 39	79.0 (21.0), *n* = 43	<0.001^*∗*^
	30 minutes after ROSC	128.0 (15.2), *n* = 7	99.5 (14.2), *n* = 4	0.013^*∗*^
	60 minutes after ROSC	121.9 (12.5), *n* = 7	107.0 (NA), *n* = 2	NA

MAP (mmHg)	After CPR	60.0 (11.2), *n* = 39	67.3 (9.9), *n* = 43	0.003^*∗*^
	30 minutes after ROSC	51.8 (14.4), *n* = 7	60.0 (9.1), *n* = 4	0.338
	60 minutes after ROSC	53.8 (8.3), *n* = 7	65.0 (19.8), *n* = 2	0.567

Respiration rate (breaths/min)	After CPR	18.7 (10.6), *n* = 13	20.3 (5.6), *n* = 12	0.631
	30 minutes after ROSC	21.3 (0.5), *n* = 4	20.7 (1.5), *n* = 3	0.582
	60 minutes after ROSC	26.5 (3.1), *n* = 4	21.0 (NA), *n* = 2	0.078

Data are presented as mean (standard deviation) or number and percentage.

ALP-CPR, abdominal lifting and compression cardiopulmonary resuscitation; BMI, body mass index; MAP, mean arterial pressure; NA, not available; ROSC, return of spontaneous circulation; STD-CPR, standard CPR.

^*∗*^ *P* < 0.05 indicates a significant difference between ALP-CPR and STD-CPR groups.

**Table 4:** Associations of sex and changes of vital signs and blood gas measurements after ALP-CPR and STD-CPR.

	ALP-CPR		*P* value	STD-CPR		*P* value
	Male (*n* = 23)	Female (*n* = 17)		Male (*n* = 22)	Female (*n* = 21)	
pH	−0.17 (0.18)	−0.16 (0.18)	0.824	−0.08 (0.20)	−0.10 (0.07)	0.666
SPO_2_	21.59 (51.12)	2.74 (46.10)	0.230	12.95 (44.57)	16.09 (25.12)	0.779
PCO_2_	−3.65 (11.67)	−5.78 (11.97)	0.576	6.43 (16.51)	−3.82 (17.95)	0.059
PO_2_	13.18 (16.70)	17.26 (13.93)	0.405	−6.57 (33.04)	4.59 (25.85)	0.223
K^+^	−0.27 (1.51)	0.00 (0.92)	0.477	−0.07 (0.84)	0.03 (1.15)	0.750
Ca^2+^	0.45 (1.02)	0.25 (1.11)	0.556	0.06 (0.24)	−0.11 (0.35)	0.083
LAC	−0.52 (0.68)	−0.35 (0.41)	0.365	−1.29 (1.03)	−1.34 (1.21)	0.884
Heart rate	106.65 (9.27)	106.86 (9.60)	0.944	77.05 (24.62)	80.86 (17.23)	0.558
Respiration rate	19.33 (10.88)	18.14 (11.22)	0.850	15.33 (1.86)	25.33 (2.34)	<0.001^*∗*^
MAP	60.98 (11.63)	59.22 (11.08)	0.633	69.97 (7.77)	64.68 (11.15)	0.080

ALP-CPR, abdominal lifting and compression cardiopulmonary resuscitation; STD-CPR, standard CPR; LAC, lactate; MAP, mean arterial pressure.

^*∗*^ *P* < 0.05 indicates a significant difference between groups.

**Table 5:** Associations of age and changes of vital signs and blood gas measurements after ALP-CPR and STD-CPR.

	ALP-CPR		*P* value	STD-CPR		*P* value
	Age > 65 years (*n* = 20)	Age ≤ 65 years (*n* = 20)		Male (*n* = 22)	Female (*n* = 21)	
pH	−0.20 (0.13)	−0.13 (0.21)	0.209	−0.06 (0.19)	−0.11 (0.10)	0.351
SPO_2_	7.80 (53.17)	13.70 (44.71)	0.706	12.71 (35.01)	16.32 (36.80)	0.744
PCO_2_	−1.50 (10.28)	−8.25 (12.39)	0.068	1.14 (17.09)	1.23 (18.91)	0.988
PO_2_	12.15 (18.17)	18.90 (10.67)	0.162	−3.38 (26.84)	1.55 (32.75)	0.593
K^+^	−0.52 (1.09)	0.29 (1.19)	0.031^*∗*^	0.11 (1.10)	−0.14 (0.90)	0.414
Ca^2+^	0.50 (1.34)	0.17 (0.69)	0.333	−0.12 (0.35)	0.06 (0.24)	0.049^*∗*^
LAC	−0.44 (0.50)	−0.41 (0.59)	0.863	−1.38 (1.03)	−1.26 (1.20)	0.734
Heart rate	107.95 (9.76)	105.53 (8.95)	0.425	76.76 (18.93)	81.14 (23.03)	0.501
Respiration rate	23.86 (7.73)	12.67 (10.84)	0.053	18.43 (5.56)	23.00 (4.95)	0.173
MAP	59.19 (11.28)	60.83 (11.38)	0.654	68.76 (10.41)	65.84 (9.42)	0.339

ALP-CPR, abdominal lifting and compression cardiopulmonary resuscitation; STD-CPR, standard CPR; LAC, lactate; MAP, mean arterial pressure.

^*∗*^ *P* < 0.05 indicates a significant difference between groups.
